# Fluorescence *In Situ* Hybridization for MicroRNA Detection in Archived Oral Cancer Tissues

**DOI:** 10.1155/2012/903581

**Published:** 2012-05-13

**Authors:** Zonggao Shi, Jeffrey J. Johnson, M. Sharon Stack

**Affiliations:** ^1^Harper Cancer Research Institute, University of Notre Dame, 1234 Notre Dame Avenue, Harper Hall A212, Notre Dame, IN 46556, USA; ^2^Department of Chemistry and Biochemistry, University of Notre Dame, 1234 Notre Dame Avenue, Harper Hall A212, Notre Dame, IN 46556, USA

## Abstract

The noncoding RNA designated as microRNA (miRNA) is a large group of small single-stranded regulatory RNA and has generated wide-spread interest in human disease studies. To facilitate delineating the role of microRNAs in cancer pathology, we sought to explore the feasibility of detecting microRNA expression in formalin-fixed paraffin-embedded (FFPE) tissues. Using FFPE materials, we have compared fluorescent *in situ* hybridization (FISH) procedures to detect miR-146a with (a) different synthetic probes: regular custom DNA oligonucleotides versus locked nucleic acid (LNA) incorporated DNA oligonucleotides; (b) different reporters for the probes: biotin versus digoxigenin (DIG); (c) different visualization: traditional versus tyramide signal amplification (TSA) system; (d) different blocking reagents for endogenous peroxidase. Finally, we performed miR-146a FISH on a commercially available oral cancer tissue microarray, which contains 40 cases of oral squamous cell carcinoma (OSCC) and 10 cases of normal epithelia from the human oral cavity. A sample FISH protocol for detecting miR-146a is provided. In summary, we have established reliable *in situ* hybridization procedures for detecting the expression of microRNA in FFPE oral cancer tissues. This method is an important tool for studies on the involvement of microRNA in oral cancer pathology and may have potential prognostic or diagnostic value.

## 1. Introduction

MicroRNA refers to the category of single-stranded small noncoding RNAs that are approximately 22 nucleotides in length. More than 1500 human microRNA have been identified and registered via various approaches including high throughput screenings (http://www.mirbase.org/). As more than 30% of human of mRNAs are regulated by microRNAs, the functional impact of microRNA in physiology and pathology has yet to be fully elucidated [1]. Generally speaking, microRNA imposes its regulatory role by sequence-specific but incomplete complementary binding to its target mRNA sequences, which are usually located at the 3′ untranslated region [1, 2]. This binding may mediate the degradation of target mRNA or the inhibition of protein translation efficiency of target mRNA. However, due to the loose stringency of this kind of targeting, the exact mechanism of specific microRNA function remains undefined and is an actively addressed research topic. The function and target mRNAs of individual microRNAs cannot be reliably predicted via current bioinformatic approaches, thereby warranting continued experimental interrogation.

The investigation of microRNA expression and its functional relationship with various cancer types has instigated tremendous interest in employing such molecules as novel diagnostic or therapeutic modalities in oncology studies [3, 4]. Recent developments that show correlation between plasma microRNA levels and cancer have further increased enthusiasm for this approach due to the easy accessibility of blood serum and plasma specimens [5]. Similarly abundant in clinical settings are archived formalin-fixed paraffin-embedded (FFPE) human cancer specimens, which can provide a rich resource for investigating the relationship between microRNA expression and cancer progression. Studies on such material have the added advantage of maintaining the information content from cancer tissue morphology when *in situ* hybridization (ISH) is used to detect microRNA expression [6]. Unlike the frequently used quantitative reverse transcription polymerase chain reaction (Q-RT-PCR) method, which requires extraction of RNA, ISH retains the microscopic topological information content with respect to microRNA expression and makes it possible to be related to other factors on the same tissue section. However, formalin fixation causes cross-links between biological molecules, potentially limiting access to microRNA molecules. Moreover, degradation has been a constant concern for preservation of RNA molecules in such tissue samples.

The *in situ* hybridization technique for nucleic acid detection on tissue and cytological preparations was initiated decades ago [7, 8], when radioisotope labeling and autoradiography were the only means to visualize positive hybridization signals. Improvement and development of newer technologies, such as the advent of nonradioactive digoxigenin as probe labeling/reporter molecule and the tyramide signal amplification (TSA) system, have made *in situ* hybridization much more accessible. However, due to the short length of microRNA molecules, *in situ* hybridization for microRNA detection remains challenging. This paper demonstrates the feasibility and highlights key technical factors of developing a protocol for fluorescence *in situ* hybridization (FISH) to detect microRNA in archived FFPE human oral cancer tissues.

## 2. Materials and Methods

### 2.1. Specimens

Formalin-fixed paraffin-embedded tissues are from lab-archived human oral cancer xenografts (10 blocks) [9] and commercially available human oral cancer tissue microarray (TMA) sections from US Biomax, Inc. (Rockville, MD), consisting of 50 tissue cores including 40 oral squamous cell carcinoma and 10 adjacent normal stratified squamous epithelia. Xenograft specimen collection was approved by the Animal Care and Use Committees (Northwestern University and University of Missouri). Surgically removed xenograft tissues were immediately fixed in 10% neutral buffered formalin for 12–24 hours and passed through dehydration, clearing, and paraffin-embedding steps. Sections were cut at 5 *μ*m thick and mounted on positively charged slides, baked at 65°C for 2 hours, and then stored at room temperature for later use. As for samples which were used to make TMA, according to the supplier, they were typically put into formalin within 15–30 minutes after surgical resection. Some of the tissue samples were snap frozen in liquid nitrogen and stored there for later fixation. Either way, fixation in neutral buffered formalin was about 24 hours before they were processed in automatic tissue processor and embedded in paraffin.

### 2.2. Reagents

Common chemical reagents were purchased from Sigma Aldrich Co. LLC. (St. Louis, MO, USA) unless otherwise specified. Normal goat serum and HRP-conjugated anti-mouse IgG were from Santa Cruz Biotechnologies, Inc. (Santa Cruz, CA, USA). Mouse antidigoxigenin and HRP-conjugated anti-digoxigenin antibodies were obtained from Roche Applied Science (Indianapolis, IN, USA). TSA-Cyanine 5 kit (NEL705A) was from Perkin-Elmer (Waltham, MA, USA), and contains HRP-streptavidin, blocking reagent, amplification diluents, and Cyanine 5-conjugated tyramide. Locked nucleic acid (LNA)-based probes were ordered from Exiqon, Inc. (Woburn, MA, USA). LNA-based microRNA miR-146a antisense oligonucleotides were labeled with digoxigenin (DIG) at the 5′ end. The human miR-146a target sequence is UGAGAACUGAAUUCCAUGGGUU, and the probe sequence is AACCCATGGAATTCAGTTCTCA. The negative control used scrambled-miR LNA detection probe that was 5′-DIG labeled. This control probe sequence is GTGTAACACGTCTATACGCCCA. LNA and non-LNA-modified 5′ biotinylated miR-146a specific probe and scrambled probe with the same sequences were custom made from Integrated DNA Technologies, Inc. (Coralville, IA, USA). ProLong mounting media containing 4′,6-diamidino-2-phenylindole (DAPI) was from Invitrogen (Carlsbad, CA, USA).

### 2.3. Special Solutions

An important consideration is to use diethylpyrocarbonate (DEPC)-treated water in all solution preparation and to exercise caution to maintain a RNase-free work environment during all procedures. To generate 20x SSC, dissolve the following in 800 mL of milli-Q grade water: 175.3 g of NaCl and 88.2 g of sodium citrate, adjust the pH to 7.0 with a few drops of 1 M HCl, and adjust the volume to 1 liter with additional distilled H_2_O. Sterilize by autoclaving. To prepare 50x Denhardt's solution, add the following to 900 mL distilled H_2_O: 10 g Ficoll 400, 10 g polyvinylpyrrolidone, and 10 g BSA, then fill up to 1 liter. Filter the solution prior to storage through a 0.2 *μ*M filter and store at 4°C (but warm up to appropriate temperature prior to use). The prehybridization solution contains the following: 50% deionized formamide, 2x SSC, 1x Denhardt's, 0.02% SDS, yeast tRNA (0.5 mg/mL), and salmon sperm DNA (0.5 mg/mL). The hybridization solution contains 50% Deionized formamide, 2x SSC, 1x Denhardt's, 10% dextran sulfate, yeast tRNA (0.5 mg/mL), and salmon sperm DNA (0.5 mg/mL).

### 2.4. Suppression of Endogenous Peroxidase

For testing the effectiveness of peroxidase inhibition, two methods (hydrogen peroxide versus hydrochloric acid) were tried simultaneously. After the sections were dewaxed and rehydrated, two kinds of fresh prepared solutions: 3% hydrogen peroxide in phosphate-buffered saline (PBS) and 0.024 M hydrochloric acid in ethanol applied, respectively, 200 *μ*L per slide, to two groups of slides and incubated for 10 minutes at room temperature. Slides were then washed with PBS (2x 5 minutes). The Cyanine 5-tyramide stock solution was diluted 1 : 50 using the included 1X amplification diluent to make the Cyanine 5-tyramide working solution. Approximately 100 *μ*L of Cyanine 5-tyramide working solution was added per slide, incubated for 10 minutes at room temperature, and washed (3 times, 5 minutes each) with PBS. Slides were then air dried in the dark and mounted using ProLong mounting media with DAPI and coverslips.

### 2.5. Fluorescence Microscopy

Solidified fluorescent slides were observed with the wide field function of an Olympus DSU spinning disc confocal microscope and SlideBook software version 4.0. Fluorescent filter sets used for DAPI are D350/50x and ET455/50m, and for Cy5 are 645/30x and ET705/72m. Pictures were taken using a Hamamatsu EMCCD camera. 

### 2.6. Procedures

**Figure 1:**
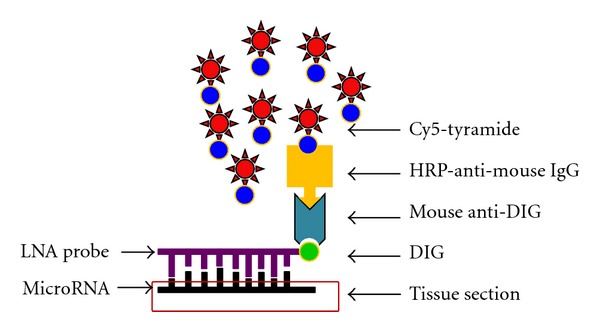
Simplified scheme of fluorescence *in situ* hybridization (FISH) for microRNA detection. Locked nucleic acid (LNA)-incorporated oligonucleotides with Watson-Crick complimentary sequence against mature microRNA was used as probe, and the 5′ end of probe was labeled with digoxigenin (DIG). Hybridization was performed at 50°C. After successful binding of such probes to their target sequence, and stringent washing steps to remove excess and nonspecifically bound probes, sequentially added were mouse anti-DIG antibody, horseradish peroxidase (HRP)-conjugated anti-mouse IgG (or HRP-anti-digoxigenin antibody in place of the 2 antibodies), and the HRP substrate Cyanine 5-conjugated tyramide. Thereafter, the positive signal could be visualized by fluorescence microscopy with proper filter sets as described in Materials and Methods section.

The typical work flow of our fluorescence *in situ* hybridization for miR-146a microRNA detection is described below. The total procedure can be completed within 2 days and is adaptable to detecting proteins of interest at the same time with multicolor labeling. A negative control slide should always be included that will be otherwise treated equally but with scrambled probe or without any probe in the hybridization step. A simplified scheme is illustrated in Figure 1.

1. Deparaffinize the Section: Bake the paraffin section at 65°C for 1 hour. Deparaffinize in xylene (2x 10 minutes), rehydrate in serial ethanol solutions (100%, 90%, 80%, 70%), and DEPC-treated water (2 minutes each) and PBS wash (2x 5 minutes).
2. Pretreatment of the Slide: Cross-linking during fixation can block reagent access to RNA/DNA molecules, so it is critical to unmask these sites using proteinases. It is also necessary to block certain endogenous active molecules that may interfere with signal development in later steps. To quench endogenous peroxidase: 0.024 M HCl in ethanol, incubate for 10 minutes, then PBS wash (2x 5 minutes). For proteinase treatment, incubate with Proteinase K (20 *μ*g/mL, 37°C for 10 minutes) followed by a PBS wash (2x 5 minutes), and complete with 4% paraformaldehyde (PFA) fixation for 10 minutes followed by a PBS wash for 5 minutes; 100 mM Glycine incubation for 10 minutes; PBS wash (2x 5 minutes) and end with a 2X SSC wash (5 minutes).
3. Prehybridization: This is meant to block nonspecific binding of probes and minimize background signals. Pre-hybridize for 2 hours at 50°C in prehybridization solution, cover with plastic coverslip, and place in a moist chamber.
4. Hybridization: Apply specific and control probes, respectively. Hybridize overnight (18 hours) at 50°C with probe at the concentration of 25 nM (1 *μ*L 2.5 *μ*M DIG labeled probe to 100 *μ*L hybridization solution), cover with plastic coverslip and place in a moist chamber.
5. Stringency Wash: This step is critical to remove nonspecific probe binding and overloaded probes. Wash with 2X SSC (37°C for 15 minutes) followed by a high temperature 2X SCC wash (at 50°C with shaking). Next wash with 1X SSC (2x at 37°C for 15 minutes), with shaking at 50°C in 1X SCC for another 15 minutes, and wash with 0.02% SDS in 1X SSC (2x at 37°C for 15 minutes), with shaking at 50°C in the same buffer for another 15 minutes. Then followed by PBS-T (PBS containing 0.1% tween-20) washes at room temperature (RT) (4x 5 minutes).
6. Posthybridization Immunohistochemistry: Use properly labeled antibody to visualize the positive hybridization signals. Serum block uses 10% normal goat serum in PBS for 1 hour at room temperature, followed by mouse anti-DIG (1 : 250) in the above blocking solution for 0.5 hour at room temperature; rinse with PBS-T wash (4x 5 minutes). Add HRP-conjugated goat anti-mouse IgG (1 : 500) for 0.5–1 hour at room temperature. Note that the use of 2 antibodies (mouse anti-DIG and HRP-conjugated goat anti-mouse IgG) could be replaced with HRP-conjugated anti-DIG only. Following antibody incubation, wash with PBS-T (4x 5 minutes) and add Cy5-tyramide working solution (100 *μ*L per slide); incubate at room temperature for 10 minutes. PBS-T wash (4x 5 minutes); PBS wash (5 minutes), air-dry for 10 minutes in the dark, and apply ProLong Gold mounting medium with DAPI and a coverslip. After overnight solidification, slides are ready for observation with fluorescent microscopy.

## 3. Results and Discussion

The need to further evaluate the expression and function of specific microRNAs in cancer pathology prompted us to establish a method of detecting microRNAs in archived FFPE materials. Abundant concerns regarding the integrity of RNA in FFPE samples have been confirmed by studies showing that mRNA in FFPE materials has various degrees of degradation as well as chemical modification by the fixative, rendering downstream analysis of extracted RNA difficult [10]. Interestingly however, recent studies using microarray analyses to compare RNA species between paired FFPE and fresh frozen samples demonstrated closely related microRNA profiles, while mRNA profiles exhibited signs of degradation in FFPE materials [11, 12]. Time to fixation, nuclease activity before fixation, chemical modification by formalin and variation in sample-processing procedures likely contribute to the decline in mRNA quality in FFPE relative to fresh frozen tissue samples. However, as demonstrated in the aforementioned studies, the small size of microRNA as well as its close association with large protein complexes enable the relatively better maintenance of their integrity after formalin fixation and paraffin embedding. This high degree of correlation between microRNA signatures in FFPE and fresh frozen specimens encourages further exploration of microRNA detection methods on archived pathology samples. After optimizing certain critical conditions, we have found that LNA-based probes labeled with digoxigenin and combined with TSA amplification provided satisfactory results for miR-146a FISH detection in archived oral cancer tissues. These results and conditions are discussed below. Specimen processing in our study was relatively well controlled; we have to acknowledge that results on specimens from real-life clinical archives may exhibit greater variability. 

### 3.1. Endogenous Peroxidase Is Better Inhibited by Hydrochloric Acid Solution

**Figure 2:**
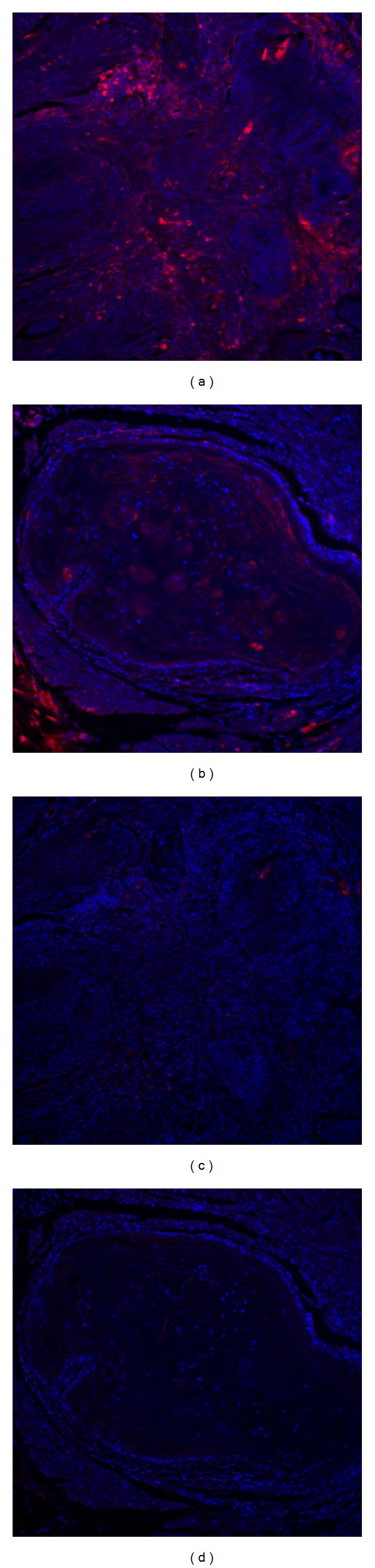
Suppressing endogenous peroxidase is critical for TSA-based *in situ* hybridization. Residual peroxidase activity from endogenous sources leads to background staining and obscures the truly positive signals. Hydrogen peroxide (H_2_O_2_) and hydrochloric acid (HCl) solution were compared for effectiveness in suppression of endogenous peroxidase activity. (a, b) Incubation with high concentration (3%) H_2_O_2_ in PBS (instead of more commonly used 0.3% H_2_O_2_) for 10 minutes does not suppress endogenous peroxidase. (c, d) The suppression from 0.024 M hydrochloric acid in ethanol for 10 minutes is more extensive. Red: Cy5-tyramide showing existence of peroxidase activity, blue: 4′,6-diamidino-2-phenylindole (DAPI) stained nuclei. Original magnification 200x.

Nonradioactive *in situ* hybridization techniques often rely on immunohistochemistry to amplify positive hybridization signals. Horseradish peroxidase (HRP) conjugated antibodies combined with chromogenic substrates are frequently employed for this purpose. However, some tissues and cells contain endogenous peroxidase, especially leukocytes and erythrocytes. In cancer tissues, because of abundant angiogenesis and frequent inflammatory infiltration, peroxidase-containing cells are common. This is a major concern when using the highly sensitive TSA system, the mechanism of which depends on peroxidase activity and which provides up to 1000-fold amplification in detection sensitivity [13], as endogenous peroxidases produce significant background staining when not inhibited properly. Because of this concern, we tested different quenching methods for endogenous peroxidase. Hydrogen peroxide is mostly commonly used in immunohistochemistry, at a typical concentration from 0.3% to 3% diluted in methanol or PBS buffer and an incubation time of 10 to 60 minutes (lower concentrations require longer incubation times but may induce less damage to certain antigens of interest). A less known method was reported by Weir and colleagues decades ago, that is, to incubate slides with 0.024 M hydrochloric acid (HCl) in ethanol for 10 minutes to efficiently destroy endogenous peroxidase [14]. We compared the use of 3% H_2_O_2_ (Figures 2(a) and 2(b)) and 0.024 M HCl (Figures 2(c) and 2(d)) on consecutive FFPE sections. The difference is striking, wherein incubation with the HCl solution produced a nearly complete suppression of endogenous peroxidase when incubated only for 10 minutes, but in 3% H_2_O_2_-treated samples at the same incubation time, inflammatory cells and even some carcinoma cells are quite positive with highly sensitive Cy5-tyramide detection (Figure 2).

 Complete suppression of endogenous peroxidase activity may not be necessary for routine immunohistochemistry as trace peroxidase will not detectably affect the chromogenic reaction. However, with the tyramide signal amplification system, trace amounts of active peroxidase can result in tyramide precipitation, and this high sensitivity dictates the needs for thorough inhibition of such enzyme activity. As demonstrated (Figure 2), dilute HCl solution is a convenient, low cost, and highly effective replacement for the traditional H_2_O_2_-blocking step. As for the timing of peroxidase blocking, we apply this step after section rehydration and before proteinase K treatment. It should be noted that acid treatment may also aid hybridization itself. 

### 3.2. Biotin versus Digoxigenin-Labeled Probes

Properly labeled probes are the most crucial reagent for *in situ* hybridization. The ready availability of commercially labeled oligonucleotide-based probes enables end users to choose the tracer or reporter that best fits their protocols. Biotin, digoxigenin, and fluorescein have been frequently used as reporter molecules on probes. 

**Figure 3:**
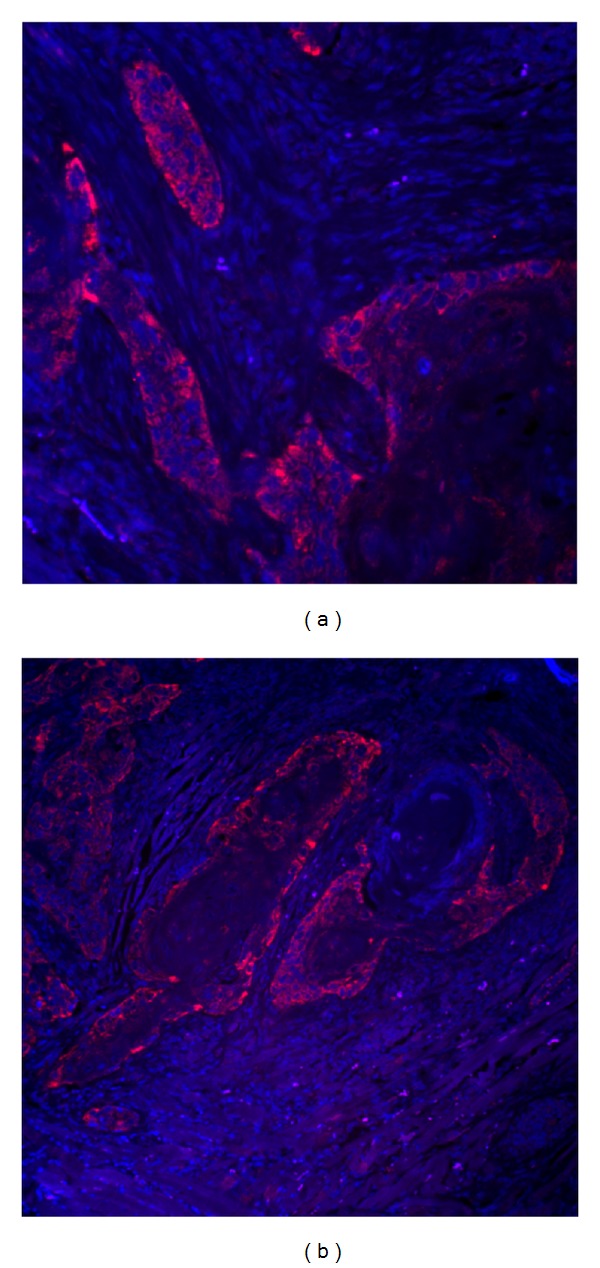
Endogenous biotin in oral cancer tissues. Following satisfactory inhibition of endogenous peroxidase using dilute hydrochloric acid solution (Figure 2), fluorescence *in situ* hybridization was performed using (a) 5′ biotinylated complementary DNA oligonucleotide probe against miR-146a or (b) control sample treated the same way but without the addition of probe (i.e., without any exogenous biotin). The control sample reveals endogenous biotin in oral cancer tissues. Detection used streptavidin-HRP and Cyanine 5-conjugated tyramide. Red: Cy5-tyramide showing existence of streptavidin-binding activity, blue: DAPI stained nuclei. Original magnification 200x.

Early versions of the commercially available TSA system used streptavidin-biotin affinity for initial signal detection. In this reaction schema, a biotinylated probe hybridizes with a single-stranded target sequence, HRP-conjugated streptavidin binds to the biotin, HRP catalyzes the activation of tyramide conjugated with fluorescein, and active tyramide precipitates in the vicinity of HRP molecule. Based on this protocol, we initially used biotinylated probes for *in situ* hybridization. However, exploratory tests confirmed that this is not appropriate for the type of tissues evaluated, as oral cancer cells are rich in endogenous biotin (Figure 3). Indeed endogenous biotin (or a similar streptavidin-binding activity) has been reported in many types of human tissues. To circumvent this nonspecific staining problem, alternative reporters are required. Digoxigenin (DIG) has been long proven to be a good option as reporter molecule in nucleic acid probe design [15–17]. As a heptan, the only natural source of DIG is digitalis plants, significantly reducing the likelihood that the anti-DIG antibody will bind any endogenous antigens in animal tissues. This provides the advantage of less nonspecific background in *in situ* hybridization.

### 3.3. LNA Probes Provide Better Specificity and Sensitivity

Locked nucleic acid (LNA) is a nucleic acid analog that contains at least one nucleotide monomer with a bicyclic furanose ring locked in a conformation mimicking RNA [18]. This configuration is advantageous as a probe material for microRNA detection. As the length of microRNA is only about 22 nucleotides, many microRNA molecules are differentiated from each other by only a few bases, such that it is difficult to achieve appropriate high-level probe sensitivity and specificity. The physicochemical properties of LNA provide an effective solution to this problem. LNA-modified oligonucleotides demonstrate much higher thermal stability and higher melting temperatures when hybridized with target RNA sequences compared to unmodified counterparts [19]. It improves the mismatch discrimination, increasing the base-pairing selectivity and providing the needed high degree of affinity for effective microRNA hybridization.

**Figure 4:**
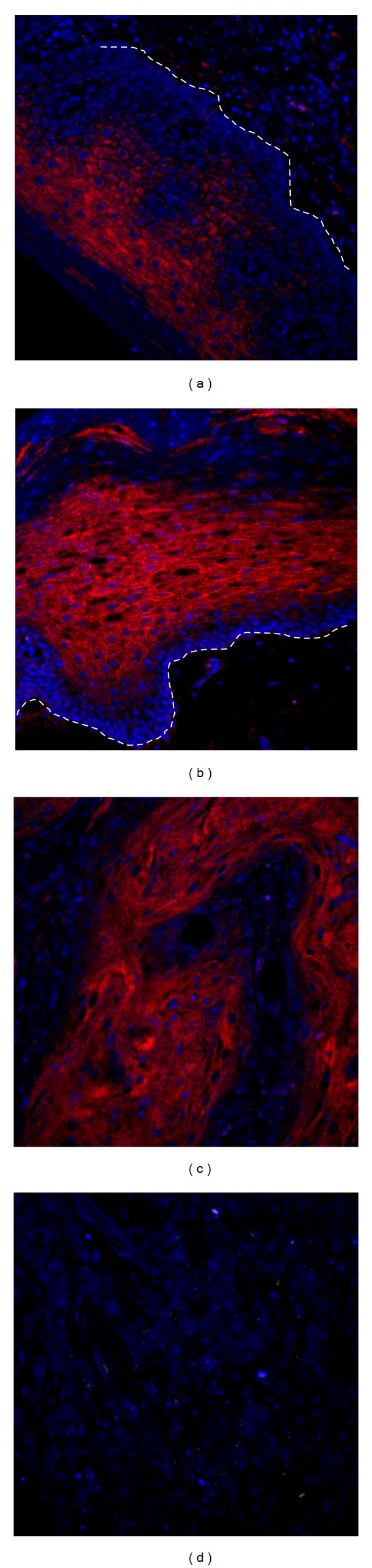
miR-146a expression in oral normal squamous epithelia and squamous carcinoma. Microarrayed normal oral tissue (10 cores) and oral squamous cell carcinoma (40 cores) were evaluated using the optimized fluorescence *in situ* hybridization protocol as outlined in Figure 1. The probe for miR-146a is LNA modified and labeled with digoxigenin at 5′ end. (a, b) Normal oral squamous epithelia. As shown in (a) and (b), cells at the basal layer (adjacent to the white dashed line) are largely negative for miR-146a, while cells at the intermediate layer are positive. In oral squamous carcinomas, (c) well-differentiated tumors, shown here is tissue core B6, often exhibit positive miR-146a staining while (d) poorly differentiated tumors, shown is tissue core E8, tend to be negative. Red: Cy5-tyramide showing positive hybridization signals, blue: DAPI-stained nuclei. Original magnification 400x.

 Using a commercially available LNA-modified DIG-labeled probe under the conditions outlined above resulted in appropriate differential staining (Figure 4). Prickle cells (stratum spinosum) in most normal oral squamous epithelia were positive for miR-146a, while basal cells exhibited negative staining. This kind of good performance is consistent with other studies showing the use of LNA-based probes for hybridization-based microRNA detection [19].

### 3.4. Tyramide Signal Amplification System Provides Enhanced Sensitivity

The introduction of tyramide conjugates as substrates for HRP has revolutionized the sensitivity of any HRP-based detection system [20]. Mechanistically, HRP reacts with hydrogen peroxide and the phenolic part of tyramide and produces a quinone-like structure with a radical on the C2 group, becoming “activated.” Activated tyramide then rapidly and covalently binds to all nearby tyrosine residues with proximity to the initially immobilized HRP site such that signal resolution is not compromised [21]. Previous studies have shown that the tyramide signal amplification system, which was also known as catalyzed reporter deposition method (CARD), provides the capability to identify single copy DNA or RNA with conjugated fluorophore [21, 22].

To detect microRNA *in situ* with probe-based approaches requires such highly sensitive signal amplification. We have tested the use of the more traditional chromogenic substrate diaminobenzidine (DAB) with HRP-labeled anti-DIG antibody in the context of LNA-based DIG-labeled probes but did not get well-differentiated staining in *in situ* hybridization. The use of TSA eliminated this difficulty. The caveat is that when using any TSA system, complete suppression of endogenous peroxidase is essential, as discussed above.

Another well-recognized way of amplifying positive signal from nucleic acid hybridization is branched DNA signal amplification [23, 24], which makes use of multiple layers of probes with the last layer being extensively enzyme labeled. However, for microRNA detection, the primary probes still require the LNA or LNA-like nucleotides to enhance the initial specificity and sensitivity, and signal amplification with the branched DNA procedure is less customizable than the TSA system allows. The use of branched DNA signal amplification for *in situ* hybridization microRNA detection has not been reported.

## 4. Conclusion

FFPE tissues initially appeared to be a challenging platform for microRNA detection but are actually better suited for microRNA than mRNA studies as recently revealed [10, 11, 25]. Thus, clinically archived cancer tissue specimens can represent buried treasure, as microRNAs are well preserved in such materials. Our study has demonstrated that after efficient inhibition of endogenous peroxidase, LNA-based and digoxigenin-labeled probe, applied together with tyramide signal amplification, significantly improves the results of fluorescence *in situ* hybridization for microRNA detection.

In summary, we have established a feasible *in situ *hybridization procedure for detecting the expression of microRNA in FFPE oral cancer tissues. This detection is important for studies on the participation of microRNA in oral cancer pathology and may have potential prognostic or diagnostic value as large cohort studies using such material will confirm.
